# Obesity is a risk factor for central precocious puberty: a case-control study

**DOI:** 10.1186/s12887-021-02936-1

**Published:** 2021-11-16

**Authors:** Gaiyan Liu, Jinxin Guo, Xuejing Zhang, Yu Lu, Junjie Miao, Hongmei Xue

**Affiliations:** 1 Department of pediatrics, Xingtai Third Hospital, Xingtai, P.R. China; 2 Key Laboratory of Public Health Safety of Hebei Province, College of Public Health, Hebei University, Baoding, 071002 China; 3 School of Public Health, Hebei Medical University, Shijiazhuang, 050017 China; 4 Department of Clinical Nutrition, The First Hospital of Hebei Medical University, Shijiazhuang, 050031 Hebei China

**Keywords:** Central precocious puberty, Overweight, Obesity, Case-control study, Puberty onset

## Abstract

**Background:**

Obesity is an important underlying cause of central precocious puberty (CPP), but previous large studies are flawed by using just age and breast examination to diagnose CPP. We aimed to determine whether overweight and obesity in childhood increases hormonally diagnosed CPP.

**Methods:**

Our retrospective, case-control study recruited 846 children diagnosed as having CPP and randomly sampled 1650 healthy control subjects in Xingtai Third Hospital in China between November 2018 and March 2021. Information was obtained from an electronic medical record and questionnaire investigated in the outpatient visit. Observations were made before the a priori hypothesis. Unconditional logistic regression for analysis was used to determine whether overweight and obesity status and duration of overweight/obesity were associated with CPP.

**Results:**

Overweight and obesity were significantly associated with increased odds of CPP among girls, even after adjusting for birth weight, exclusive breastfeeding for 6 month, household income, maternal overweight, paternal overweight, and maternal menarche age (overweight: the adjusted odds ratio (aOR) (95%CI): 1.92 (1.16, 3.24), *p* = 0.02; obesity: aOR (95%CI): 1.78 (1.13, 3.48), *p* = 0.03). Furthermore, the effects of overweight and obesity were significant when ongoing for 1 to 2 years, 2 to 3 years, and greater than 3 years, but not at less than 1 year. For boys, association between obesity and increased odds of CPP was observed (aOR (95%CI): 1.68 (1.09, 3.75), *p* = 0.03). The effects of overweight and/or obesity were only significant when ongoing for greater than 2 years.

**Conclusions:**

Prolonged overweight and obesity in early childhood may be risk factors for CPP, especially in girls. Weight loss might be an important approach for the prevention of precocious puberty in children.

## Background

Central precocious puberty (CPP) is a disease of abnormal growth and development, characterized as early onset of adolescent secondary sexual characteristics resulting from early commencement of pulsatile secretion of gonadotrophin releasing hormone (GnRH) [1]. CPP can threaten the physical and mental health of children, and may be a significant cause of many kinds of chronic diseases in adulthood (insulin resistance, obesity, cancer of the reproductive system etc.) [2–4]. Observational data [5] from the US [6], Europe [7], Denmark [8], Korea [9] and China [10] showed that the prevalence of CPP has increased significantly in recent years, up to 0.5–2% in China. The underlying causes for the increase in the rate of CPP are being studied. A number of evidence found that the rate of obesity has increased by more than double in children and adolescents in parallel with the rise in CPP [11, 12].

Obesity has been well documented to be a very important risk factor for metabolic disease [13]. However, whether CPP in adolescents is related to obesity is an ongoing topic for debate. Epidemiological cross-sectional and longitudinal studies show a shift towards earlier onset of puberty in girls who are obese, which may be due to girls’ higher body fat mass [14, 15]. But, limited research has been conducted among boys. Moreover, it is worth noting that most epidemiological studies diagnosed puberty onset through clinical features only, breast development reached Tanner II stage before age 8 in girls and testicular volume was ≥4 mL before age 9 in boys [1, 16]. But, breast development or testicular enlargement is not synonymous with CPP, the latter of which need to be diagnosed by the GnRH provocation test (the gold standard) [17–19]. We are not aware of any research looking at the association between obesity and hormonally diagnosed CPP.

To fill this gap, we conducted a retrospective, unmatched case-control study sampled a number of 846 children (681 girls and 165 boys) with CPP and 1650 randomly sampled healthy control subjects (1290 girls and 360 boys) who ever visited the pediatrician’s or endocrinologist’s office in Xingtai Third Hospital in China between November 2018 to March 2021, to determine the effect of obesity on CPP.

## Methods

### Study sample

Children diagnosed as having CPP in Xingtai Third Hospital in China between November 2018 and March 2021 was all recruited in the present study. The inclusion criteria for case subjects were as follows: a) children who have done clinical diagnostic examination and diagnosed by a professional pediatrician, and b) male or female sex. Exclusion criteria included: a) diagnosis of CPP before diagnosis of overweight status or obesity, b) related to increased sex steroid production independent of GnRH, c) history of metabolic disease affecting overweight or obesity status and d) has taken hormone-related medications before. A number of 846 children with CPP were included for the study (15 were excluded because of incomplete follow-up, and 34 were excluded based on exclusion criteria).

Control subjects were randomly selected from the electronic medical record of outpatient pediatric practices in the same hospital and same period of children diagnosed with CPP (n = approximately 30, 000–40, 000 patient pool) using a computerized random number generators by a statistician who was not involved in the data collection. Those who have history of metabolic disease affecting overweight or obesity status and have taken hormone-related medications before were excluded. A total of 1650 control subjects without a history of CPP who had complete follow-up and met the inclusion criteria were randomly selected as control subjects in the present analysis. The ratio of case to control is almost 1: 2 among girls and boys, respectively. This study was approved by the Ethics Committee of Xingtai Third Hospital (Approved number: 2020-KY-40) and adhere to the Helsinki Declaration in the course of implementation. All children and their parents gave written informed consent.

### Central precocious puberty

Criteria for the diagnosis of CPP were based on the guidelines formulated by Ministry of Health of the People’s Republic of China in 2011 and Chinese Medical Association in 2015 [17, 18], which are as follows: a) Earlier occurrence of secondary sexual characteristics (age within definition: the age of 9 and the age of 8 when girls have secondary sexual characteristics), and which follow the normal developmental process; b) Bone age ahead: Bone age 1 year or more over chronological age; c) There is evidence of gonadal development, the girls were judged by B-mode ultrasound, and the testicular volume of the boy was ≥4 mL; d) There is a sudden increase in height during development. e) Function of hypothalamic-pituitary-gonadal axis (HPGA) started and gonadotropins are elevated to pubertal levels: 1) Unstimulated luteinizing hormone (LH) detected by immunochemiluminometric assays > 5.0 U/L; “or” 2) when unstimulated LH is between 0.1 and 5.0 U/L, GnRH provocation test was conducted: the excitation peak value of LH > 5.0 U/L “and” LH / FSH (Follicle Stimulating Hormone) > 0.6 by GnRH provocation test was diagnosed as CPP. When the first four conditions (a), b), c), d)), and one of two conditions 1) or 2) are met, CPP was diagnosed.

### Anthropometric measurements

Anthropometric measurements were routinely performed according to standard procedures by a physician at each outpatient visit, with the subjects dressed lightly and barefoot. An Ultrasonic Weight and Height Instrument was used to assess the standing height and weight to the nearest 0.1 cm and 0.1 kg, respectively. All anthropometric measurements were performed twice with the average calculated for each participant. In addition, weight and height status over the past years like “How many years have you been overweight or obesity?” were also answered by the parents.

Body mass index (BMI) was calculated as follows: weight/height^2^. The overweight and obesity thresholds for sex-age-specific BMI screening in school-age children and adolescents were used to define children overweight and obesity (cut-offs of P85 for overweight and P95 for obesity) [20]. It was hypothesized that long-term, rather than short-term, overweight/obesity is a stronger risk factor for CPP. Therefore, duration of overweight and obesity status was encoded as ordinal categorical variable representing less than 1, 1 to 2, 2 to 3, and greater than 3 years, respectively.

### Covariates

Information on children’s birth characteristics, feeding practices, and social-demographic data was provided by parents when they visit the doctors, which as follows: a) birth characteristics and feeding practices including fetus age (in weeks), birth weight (in kg), birth length (in cm), exclusive breastfeeding duration (in month), and timing of complementary feeding; b) social-demographic data including place of residence, household income (< 15,000 RMB, 15,000–35,000 RMB and > 35,000 RMB), parental age (in years), and parental occupation (liberal profession, casual laborer, manual worker, and non-manual worker) and education levels (< 6 years, 6–12 years, and > 12 years of schooling). Additionally, parents’ height (in cm) and weight (in kg) were self-reported, and information on maternal menarche age (in years) was also collected.

### Statistical analysis

SAS 9.4 software (SAS Institute Inc., Cary, NC, USA.) was used for statistical analysis. A significance level at *p* < 0.05 was taken to indicate statistical significance for all estimates. Normality of all continuous variables was examined by using normal probability plots and the Kolmogorov-Smirnov test. Given their non-normality, all continuous variables were presented as median (25th percentile, 75th percentile). Significant differences for non-normally distributed continuous variables between case group and control group were analyzed by Wilcoxon rank sum test, and chi-square tests were used for categorical variables. All the analyses in this study were performed for girls and boys separately.

Unconditional logistic regression for analysis was used to determine whether overweight and obesity status, and duration were associated with CPP. Potential covariates that may affect these associations like children’s birth characteristics, feeding practices, and social-demographic data were included in the models. Household income, parental occupation, parental education level, parental overweight (BMI ≥ 24 kg/m^2^) [21], exclusive breastfeeding for 6 months were modeled as categorical variables. Timing of complementary feeding, parental age, and mother’s menarche age were modeled as continuous numerical variables. Unconditional logistic regression was also used to determine whether duration of overweight and obesity status were associated with CPP. For duration of overweight and obesity, the independent variable was encoded as an ordinal variable from 0 to 4 representing normal weight and overweight/obesity (< 1, 1–2, 2–3, and > 3 years).

The odds ratio (OR) for CPP and its 95% confidence interval (CI) were used to represent the relative risk. The adjusted OR (aOR) was calculated by including birth weight, exclusive breastfeeding duration, household income, maternal overweight, paternal overweight, and maternal menarche age in the model.

## Results

Comparison of baseline characteristics in case group and control group are presented in Table 1. The ratio of case to control is almost 1: 2 among girls and boys, respectively. During data collection, nearly four fifths CPP patient were girls. Age did not differ between case and control groups (*p* = 0.5). Girls with CPP had lower rates of exclusive breastfeeding for 6 month, higher birth weight, higher overweight and obesity rates, and whose mother and father had higher overweight rates than those of control group (*p* < 0.03). For boys, children in case group had lower rates of exclusive breastfeeding for 6 month, higher overweight and obesity rates compared with boys in the control group (*p* < 0.03). No difference of other birth characteristics and social-demographic data between boys with and without CPP (*p* > 0.1).

**Table 1:** Comparison of baseline characteristics in case group and control group^a^

Characteristics	Case	Control	*P* value
Girls			
n (%)	681 (34.55)	1290 (65.45)	–
Age, years	7.02 (6.21, 7.84)	7.06 (6.30, 7.91)	0.5
Fetus age, weeks			
≤ 37	13.72	12.84	0.3
38–40	69.81	73.62	
≥ 41	15.56	13.69	
Birth weight, kg	3.22 (2.90, 3.52)	2.61 (2.23, 3.46)	0.03
Breastfeeding for 6 months (%)	52.42	68.68	< 0.0001
High household income level ^b^ (%)	24.67	23.64	0.6
High maternal education level ^c^ (%)	25.70	22.40	0.1
High paternal education level ^c^ (%)	26.28	25.66	0.8
Maternal overweight ^d^ (%)	45.37	38.14	0.002
Paternal overweight ^d^ (%)	47.28	40.70	0.005
Maternal menarche age	12.31 (11.03, 14.17)	12.82 (11.53, 14.42)	0.06
Overweight ^e^ (%)	16.89	9.07	< 0.0001
Obesity ^e^ (%)	12.28	3.49	< 0.0001
Boys			
n (%)	165 (31.43)	360 (68.57)	–
Age, years	8.22 (7.54, 8.83)	8.23 (7.67, 8.90)	0.6
Fetus age, weeks			
≤ 37	13.89	12.37	0.4
38–40	69.74	73.10	
≥ 41	16.37	14.53	
Birth weight, kg	3.42 (2.89, 3.98)	3.37 (2.75, 3.81)	0.1
Breastfeeding for 6 months (%)	58.18	68.89	0.02
High household income level ^b^ (%)	24.24	26.94	0.5
High maternal education level ^c^ (%)	18.79	24.17	0.2
High paternal education level ^c^ (%)	24.85	25.28	0.9
Maternal overweight ^d^ (%)	41.82	37.22	0.3
Paternal overweight ^d^ (%)	43.64	41.39	0.6
Maternal menarche age	12.52 (11.01, 14.24)	12.73 (11.14, 14.45)	0.1
Overweight ^e^ (%)	20.61	14.44	0.03
Obesity ^e^ (%)	14.18	8.34	0.02

^a^ Values are median (25th percentile, 75th percentile) or frequencies. Test for difference between boys and girls was performed by using Wilcoxon rank-sum for non-normally distributed continuous variables and chi-square test for categorical variables

^b^ Average family income more than 35,000 RMB every year;

^c^ At least 12 years of school education

^d^ BMI (in kg/m^2^) ≥24 [21]

^e^ Calculated according to the Working Group on Overweight and Obesity in China criteria [20]

The effects of overweight and obesity status on CPP by using unconditional logistic regression analysis are presented in Table 2. Overweight and obesity were associated with increased odds of CPP among girls after control for confounding factors like birth weight, exclusive breastfeeding for 6 month, household income, maternal overweight, paternal overweight, and maternal menarche age (overweight: OR (95%CI): 1.92 (1.16, 3.24), *p* = 0.02; obesity: OR (95%CI): 1.78 (1.13, 3.48), *p* = 0.03). For boys, association between obesity and increased odds of CPP was observed, even after adjustment for the covariates (aOR (95%CI): 1.68 (1.09, 3.75), *p* = 0.03). We found a trend but not a statistically significant effect of overweight on CPP in boys (*p* = 0.09).

**Table 2:** Unconditional logistic regression analysis of the effects of overweight status and obesity on central precocious puberty

	No. Of participants (n, %)		Central precocious puberty			
	Case	Control	OR (95%CI)	*P* value	aOR^a^ (95%CI)	*P* value
**Girls**						
Normal weight	482 (70.83)	1128 (87.44)	1	–	1	–
Overweight	115 (16.89)	117 (9.07)	2.30 (1.74, 3.04)	< 0.0001	1.92 (1.16, 3.24)	0.02
Obesity	84 (12.28)	45 (3.49)	2.09 (1.74, 2.53)	< 0.0001	1.78 (1.13, 3.48)	0.03
**Boys**						
Normal weight	108 (65.45)	278 (77.22)	1	–	1	–
Overweight	34 (20.61)	52 (14.44)	1.68 (1.03, 2.73)	0.04	1.02 (0.94, 3.16)	0.09
Obesity	23 (13.94)	30 (8.34)	1.41 (1.04, 1.88)	0.02	1.68 (1.09, 3.75)	0.03

^a^ aOR was calculated by including birth weight, exclusive breastfeeding for six month, household income, and maternal overweight, paternal overweight, and maternal menarche age in the model

Furthermore, The effect of duration of overweight and obesity on the OR of CPP was determined by unconditional logistic regression models using dummy variable coding of intervals for years of obesity (< 1 years, 1–2 years, 2–3 years, and > 3 years), which is resented in Table 3. Among girls, children with overweight and obesity for 1 to 2 years, 2 to 3 years, and greater than 3 years were at high risk for CPP, almost 1.65, 2.01 and 2.48 times than normal weight children, respectively (1 to 2 years: aOR (95%CI = 1.65 (1.10, 2.77), *p* = 0.01; 2 to 3 years: aOR (95%CI = 2.01 (1.54, 2.86), *p* = 0.0002); greater than 3 years: aOR (95%CI) = 2.48 (1.67, 3.72), *p* = 0.0001). For boys, the effects of overweight and obesity were only significant when ongoing for greater than 3 years (aOR (95%CI): 2.10 (1.24, 3.68), *p* = 0.01). A bordline effects of overweight and obesity were found when ongoing for 2 to 3 years (aOR (95%CI): 2.23 (1.00, 2.53), *p* = 0.047). These data suggest that prolonged overweight and obesity increases CPP in girls, and may also in boys.

**Table 3:** Unconditional logistic regression analysis of the duration of overweight and obesity status on central precocious puberty

	No. Of participants (n, %)		Central precocious puberty			
	Case	Control	OR (95%CI)	*P* value	aOR^a^ (95%CI)	*P* value
Girls (years)						
< 1	16 (1.91)	30 (2.32)	1.11 (0.59, 2.04)	0.7	1.08 (0.41, 2.12)	0.3
1–2	32 (4.70)	36 (2.79)	1.74 (1.15, 2.63)	0.008	1.65 (1.10, 2.77)	0.01
2–3	88 (12.92)	51 (3.95)	2.08 (1.68, 2.58)	< 0.0001	2.01 (1.54, 2.86)	0.0002
> 3	63 (9.25)	45 (3.49)	2.55 (1.88, 3.52)	< 0.0001	2.48 (1.67, 3.72)	0.0001
Boys (years)						
< 1	5 (3.03)	15 (4.17)	0.70 (0.26,1.52)	0.4	0.43 (0.18, 153)	0.7
1–2	14 (8.48)	20 (5.56)	0.98 (0.56, 1.60)	0.9	0.74 (0.41, 1.68)	0.9
2–3	20 (12.12)	24 (6.67)	2.59 (1.03, 2.47)	0.04	2.23 (1.00, 2.53)	0.047
> 3	18 (10.91)	23 (6.39)	2.23 (1.45, 3.52)	0.0004	2.10 (1.24, 3.68)	0.001

^a^ aOR was calculated by including birth weight, exclusive breastfeeding for six month, household income, maternal overweight, paternal overweight, and maternal menarche age in the model. Number of normal weight participants (*n* = 482) were not presented in the table

## Discussion

The present study suggests a relationship of overweight and obesity to CPP, independent of birth weight, exclusive breastfeeding for 6 month, household income, maternal overweight, paternal overweight, and maternal menarche age. We found that overweight and obesity among girls are related to increased odds of CPP. And these CPP-predisposing effects are found when overweight/obesity were prolonged for greater than 1 year for girls and 2 years for boys.

Albeit many studies focused on precocious puberty have been conducted by researchers, the causes in most cases in girls remain unknown. Most epidemiology studies from US [22–25], England [26], Germany [27, 28], Danmark [8], Brazil [29], Italy [30], and China [31, 32] et al. have confirmed the positive association between obesity and earlier attainment of puberty for girls. Our results are consistent with the prior studies and suggested that, among girls, overweight and obesity were significantly associated with increased odds of CPP, independent of children’s birth characteristics, feeding practices, and social-demographic data. The risks for overweight or obese children are almost twice as likely to develop CPP as normal weight children. Moreover, what is noteworthy is that, most epidemiological studies conducted in girls use breast development to determine puberty development, breast development reached Tanner II stage before age 8 in girls being identified as early age of puberty onset. But for girls, breast development is not the same as being precocious puberty. In 2013, Zhu et al. [33] conducted a survey among six areas in China showed that 2.91% of girls aged 8 years old had breast development, while after undergoing further GnRH stimulation, the actual prevalence of CPP was only 0.48%. In other words, more than 80% of the girls with breast development before the age of 8 who were found to have developed breasts during physical examination in this study were simply prematurely developing breasts. Therefore, children with precocious breast development detected should be further diagnosed in pediatric endocrinology. Compared with previous studies, our study not only used secondary sexual characteristics evaluated by Tanner stage to determine whether puberty started, but also combined with the gold standard - GnRH provocation test to further diagnose CPP. Therefore, confusion of breast and fat is much less of an issue in our study than in most others, and the present study is more convincing for confirming the relationship between obesity and precocious puberty. A larger sample size and more rigorous longitudinal study are needed to further explain the relationship between obesity and CPP.

To date, study of puberty development and its related factors among boys is limited and still a matter of controversy [34]. Some of existing researches have suggested that the effects of overweight on puberty development of boys are different from the obesity effects, showing that the puberty development of overweight boys was earlier, while that of obese boys is later [35]. And some of studies showed that the odds of puberty onset and risk of early pubertal timing were significantly increased in both overweight and obesity boys [31, 36]. .In the present study, we found that obesity rather than overweight was risk factor for CPP among boys, which is consistent with a previous study conducted among Chinese boys [37]. The reason for this phenomenon may be that the increase in body mass of adolescent boys is mainly related to the increase in bone and muscle mass [38].

Additionally, many studies [39–41] have shown that exclusive breast feeding for more than 6 months was protective for both obesity and early onset of puberty [42]. While, in the present study the association between overweight/obesity and CPP remains significant when we considered the breastfeeding as the confounding factor. Relationship of Breastfeeding, overweight and puberty is complex and needs the large-scaled cohort study to further examine.

The mechanisms of obesity and adolescent development are complex and still being studied. Therinto, some studies have found that the early onset of puberty in overweight and obese children may be linked to higher leptin levels and lower adiponectin levels, which could stimulate sex hormone production [43]. And some of studies have also shown that increased adipose tissue leads to insulin resistance, thereby reducing the concentration of sex hormone binding proteins and increasing the bio-availability of sex hormones [44].

Particularly interesting, our results also showed that prolonged overweight and /or obesity, for 1 year or more among girls, and more than 2 years among boys, can affect CPP. This suggests that reversal of overweight and obesity, if caught early, by means of aggressive weight loss intervention might be an important strategy for the prevention of CPP. It’s worth noting that, though the numbers with a duration of obesity of less than 1 year are smaller, the risk of CPP in patients in that group appears to be much lower than for those with a longer duration of obesity. Whether a short period of obesity affects the onset of puberty remains to be studied. Further studies are needed to determine whether reversal of overweight and obesity among preschoolers can improve increasing incidence of precocious puberty.

The present study had several considerable strengths, including a well-powered sample size of cases and control subjects, the high statistical efficiency, the use of multiple statistical methodologies, and the gold standard (GnRH provocation test) being used to diagnose the CPP, and other clinical information being obtained by a physician during a clinical encounter and were not just parent or questionnaire based, which are less likely to be affected by subjective parental interpretation and recall bias.

Some potentials limitations of the present study should be mentioned. Selection bias might be more prone in a retrospective/observational study, which was addressed by recruiting all patients who meet the inclusion criteria during the study period, and using a computerized random-number generator for control selection. Because this study was retrospective, several important risk factors like dietary, nutrition and physical activity histories, stress and environmental endocrine disruptors exposures and the like that related to precocious puberty were not collected. The duration of overweight and obesity status were obtained by a questionnaire and not by a direct measure, which could cause recall bias. Additionally, we only used sex-age-specific BMI to determine overweight and obesity, and did not consider the influence of body fat status on CPP. Further prospective and high quality research should be conducted to confirm our results.

## Conclusion

Our results suggest a relationship of overweight and obesity to CPP, independent of birth weight, exclusive breastfeeding for 6 month, household income, maternal overweight, paternal overweight, and maternal menarche age. We found that overweight and obesity among children, especially in girls, are related to increased odds of CPP, and that these CPP-predisposing effects are found when overweight/obesity were prolonged for greater than 1 year for girls and 2 years for boys.

## Data Availability

The datasets used and/or analyzed during the current study are available from the corresponding author on reasonable request.

## Abbreviations

CPP: Central precocious puberty
OR: Odds ratio
GnRH: Gonadotrophin releasing hormone
BMI: Body mass index
CI: Confidence interval
LH: luteinizing hormone
FSH: Follicle Stimulating Hormone
